# The Ineffective Role of Cathodal tDCS in Enhancing the Functional Motor Outcomes in Early Phase of Stroke Rehabilitation: An Experimental Trial

**DOI:** 10.1155/2014/547290

**Published:** 2014-05-05

**Authors:** Augusto Fusco, Federica Assenza, Marco Iosa, Simona Izzo, Riccardo Altavilla, Stefano Paolucci, Fabrizio Vernieri

**Affiliations:** ^1^Clinical Laboratory of Experimental Neurorehabilitation, IRCCS Fondazione Santa Lucia, Via Ardeatina 306, 00179 Rome, Italy; ^2^Department of Neurology, Campus Bio-Medico University, Via Álvaro del Portillo 200, 00128 Rome, Italy

## Abstract

Transcranial direct current stimulation (tDCS) is a noninvasive technique that could improve the rehabilitation outcomes in stroke, eliciting neuroplastic mechanisms. At the same time conflicting results have been reported in subacute phase of stroke, when neuroplasticity is crucial. The aim of this double-blind, randomized, and sham-controlled study was to determine whether a treatment with cathodal tDCS before the rehabilitative training might augment the final outcomes (upper limb function, hand dexterity and manual force, locomotion, and activities of daily living) in respect of a traditional rehabilitation for a sample of patients affected by ischemic stroke in the subacute phase. An experimental group (cathodal tDCS plus rehabilitation) and a control group (sham tDCS plus rehabilitation) were assessed at the beginning of the protocol, after 10 days of stimulation, after 30 days from ending of stimulation, and at the end of inpatient rehabilitation. Both groups showed significant improvements for all the assessed domains during the rehabilitation, except for the manual force, while no significant differences were demonstrated between groups. These results seem to indicate that the cathodal tDCS, provided in an early phase of stroke, does not lead to a functional improvement. To depict a more comprehensive scenario, further studies are needed.

## 1. Introduction

Due to increase of life expectancy, adults are progressively more exposed to vascular diseases, as stroke [1]. In the future, stroke has been predicted to account for 6.2% of the total illness (in 2020), increasing the requests to the global healthcare systems [2]. Hence, improving and speeding up the motor recovery are actually the most important challenges for neurorehabilitation.

Many of the rehabilitative techniques use knowledge derived by neuroscience to exploit the best outcomes achievable. For many years, the central nervous system (CNS) had been described as a rigid structure. More recently, a large amount of evidence has shown a wide brain cells' activity after damage [3]. This seems to be related to the neuroplasticity of the CNS and has fundamental implications for neurologic rehabilitation in terms of outcome improvements [4–6]. Neuroplasticity is defined as the brain's ability to reorganize itself by forming new neural connections throughout the life, allowing to adapt the brain in response to situations or requests derived from the environment and/or also in response to an internal damage [7, 8]. Despite the fact that structural changes related to a reorganization of the 6 preexisting networks and the axonal sprouting have been demonstrated to occur in brain cells, the mechanisms of neuroplasticity are not completely elucidated.

In the rehabilitation of patients with stroke, the first period after acute event is crucial for the recovery due to spontaneous neuroplasticity, facilitating neurophysiological repair and cortical reorganization [9]. In fact, most of motor and cognitive improvements happen early until the six months following the event, even if it is possible to show a recovery till ten years after the injury. To enhance these mechanisms, emerging technologies are developing in rehabilitation [10].

Among these, noninvasive brain stimulations (NIBS), such as transcranial magnetic stimulation (TMS) and transcranial direct current stimulation (tDCS), are techniques gradually obtaining wider interest for being used in clinical settings [11–13]. Both TMS and tDCS have properties to elicit mechanisms similar to those observed in the neuroplasticity, although the exact mechanisms of action of NIBS remain unclear.

Even from the early studies, it was evident as tDCS was able to produce a polarity-dependent modification of cortical excitability [14, 15]. tDCS can influence the electric state of the membrane potential and modulate GABAergic and glutamatergic synapses within the cortex [16].

Even if a certain degree of clinical improvements of motor performances is achieved after tDCS in patients with stroke, very low quality evidence has been demonstrated. In fact, as the principal reviews and meta-analyses have highlighted, there are a wide variability of the patients enrolled into the studies and a disparate use of stimulation parameters, limiting the conclusions on the topic at the moment [17–19]. Nevertheless, tDCS might be an emerging tool enhancing the outcomes of rehabilitation, favoring the neuroplasticity of the brain.

There are three possible electrodes' montages for tDCS: anodal, cathodal, or dual. Anodal tDCS facilitates the depolarization of neurons and hence the neural excitability; cathodal tDCS hyperpolarizes the resting membrane potential, reducing the neuronal firing: it can be used to limit the interhemispheric inhibition when mounted on the not damaged brain hemisphere. Dual tDCS combines these two montages [20]. Previous studies on tDCS investigated the efficacy of anodal montage [21, 22], whereas more recent researches were focused on contralateral inhibition by means of cathodal settings [13, 23]. However, most of these recent studies on cathodal tDCS enrolled patients in chronic phase of stroke, whereas the subacute phase is potentially more prone to neuronal reorganization and in which the signals coming from contralateral hemisphere could play a fundamental role for driving [24] or limiting the recovery [25].

The aim of this study was to evaluate the recovery of motor skills outcomes in individuals with stroke undergoing an intensive inpatient rehabilitation and electrical brain stimulation at the early stage of rehabilitation, when neuroplastic mechanisms could be exploited at best.

## 2. Material and Methods

### 2.1. Protocol

To determine whether the use of electric stimulation at an early phase of stroke may improve the motor outcomes, a double-blind, randomized, and sham-controlled trial was performed.

Every patient admitted to our unit was screened according to the diagnosis and evaluated with regard to the inclusion/exclusion criteria reported in the following section (Participants).

After obtaining the written informed consent, patients were randomized into 2 groups based on the stimulation (treatment: cathodal tDCS; no treatment: sham tDCS). The randomization was created in accordance with a binary sequence previously generated using MATLAB R2007b Software (The Matworks Inc., USA). The experimental treatment was performed in add-on for a total period of stimulation of 10 days, carried out for 2 weeks, 5 consecutive days in each week. The application of cathodal or sham tDCS was provided immediately before the rehabilitative session in order to exploit the after-effects of the stimulation. Patients were assessed before the start of the protocol (T0), after the end of the stimulation period (T1, after 10 days from T0), one month after the end of treatment (T2, 30 days after T1), and at the end of inpatient rehabilitation (T3, depending on the clinical condition of the patients, with an estimated time range between 75 and 110 days from the event).

All evaluations were performed for both the groups: cathodal tDCS as experimental group (EG) and sham tDCS as control group (CG). The patient was blind to the type of stimulation, as well as the physician performing the assessments. An unblinded investigator administered the stimulation.

This protocol was approved by the local independent ethics committee.

### 2.2. Participants

All hospitalized patients in our division (Stroke Rehabilitation Unit) were screened per inclusion/exclusion criteria. The inclusion criteria were subjects affected by a first ever stroke, age between 18 and 83 years; an occurrence of ischemic stroke in the territory of middle cerebral artery, as revealed by a magnetic resonance or computerized tomography scan performed before the enrollment; the stroke event occurred within 30 days from the starting of the protocol; no history of moderate to severe cognitive impairment, as evaluated by a neuropsychologist. The exclusion criteria were inability to perform a motor rehabilitation training; the presence of multiple foci of ischemia or a hemorrhagic stroke; the presence in the patient's history of a previous stroke or global cerebral ischemia; a diagnosis of a major psychiatric disorders or epilepsy; a history of tumor independently from location; the presence of pacemaker; uncontrolled arrhythmias or nonstabilized hearth diseases; dementia or severe aphasia that could have compromised the collaboration for the procedures.

All enrolled patients gave written informed consent before the beginning of the experimental protocol.

### 2.3. tDCS and Rehabilitative Procedures

In the EG, tDCS was performed using cathodal montage. The session of cathodal or sham tDCS had a duration of 10 minutes. The Eldith DC Stimulator tDCS model (NeuroConn, Ilmenau, Germany) was supplied to 1.5 mA of electric intensity, by 2 gel-sponge electrodes with a surface area of 35 cm^2^ (5 cm × 7 cm), embedded in a saline-soaked solution. Consequently, we delivered a current density of 0.043 mA/cm^2^ (intensity of 1.5 mA, electrode area 35 cm^2^). Stimulation was preceded by few seconds during which the current increased gradually to the selected intensity (fade-in phase), eliciting transient sensations that disappeared in several seconds and followed by the same few seconds during which current was progressively reduced (fade-out phase). The active electrode was positioned in the primary motor cortex area in the contralateral affected hemisphere (C3′/C4′ according to the International classification system of EEG electrodes placement). The reference electrode was positioned in a noncephalic side, above the right shoulder, contralateral to the electric circuit of the heart. In this way, only the area below the active electrode was stimulated, focusing the passage of current into the selected treatment area. The system is noninvasive and has been proven safe in the literature [25].

The training session following brain stimulation was that conventionally performed as part of inpatient daily rehabilitation. The motor rehabilitation was scheduled twice a day (in the morning and in the afternoon), lasting 45 minutes for each session and focused on the recovery of the upper limb and locomotor functions. Intensity and type of exercises were tailored on patient's residual abilities. No instrumented therapy was administered in the experimental period of stimulation. To avoid the presence of fatigue, the tDCS was performed before the rehabilitative session of the morning.

### 2.4. Main Outcome Measures

To evaluate the recovery of motor functions during the inpatient rehabilitation, both instrumented measures and scales were used in order to increase the sensitivity, repeatability, and accuracy of the assessments.

For a global evaluation of the patients' condition and abilities in the daily living, Canadian Neurological Scale and the Barthel Index were administered to patients.

For the assessment of the upper limb impairments, we used 9-hole peg test, dynamometers for pinch and grasp forces, and Upper Limb Fugl-Meyer Scale.

To evaluate the locomotor abilities, we applied the Timed Up and Go Test, the 6-Minute Walking Test, 10-Meter Walking Test, the Rivermead Mobility Index, and the Functional ambulation Classification.

The Canadian Neurological Scale (CNS) is a validated scale for assessing and monitoring the neurological status of patients affected by stroke, focusing on consciousness, language, and motor functions of both limbs [26]. A total score less of 6.5 strongly predicts poor outcomes including mortality at 1 month and 1year [27].

The Barthel Index (BI) is the most used scale to measure the performance in activities of daily living (ADL) [28]. The BI is a ten-item ordinal scale that covers mobility and self-care domains; the score ranges from 0 to 100. A score of 0 indicates total dependence in ADL while a score of 100 a complete independence.

The 9-hole peg test (9HPT) is a simple tool to test the hand dexterity [29]. Patients were requested to insert 9 pegs into 9 holes of a board. The time to complete the performance was measured using a chronometer. The insertion velocity were computed as the number of holes filled in the time spent to complete the test, with the limit of 50 seconds (s); velocity was measured in pegs/s as performed in previous studies [30, 31].

Specific dynamometers (Saehan Hydraulic Hand Evaluation, model SH5003; South Korea) were used to measure the manual force. Maximum pinch and grip forces were measured in 2 attempts using both hands with the patient seated and the elbow at 90° of flexion and a neutral position of the wrist. This method is well standardized and proved to have a high test-retest reliability and it has been used in similar studies [20, 30].

The Upper Limb Fugl-Meyer Scale (UL-FM) is a validated scale for the assessment of stroke-related upper-limb motor impairment [32]. It is reliable and valid and it has been often used in rehabilitative setting for its property to be sensible also in respect of small changes in terms of motor recovery during the rehabilitation [33]. It is formed by 33 items, scored on a 3-point rating scale (unable/partial ability/near normal ability to perform).

The Timed Up and Go (TUG) is a simple test used to assess both static and dynamic mobility balance [34]. Patients were asked to rise from a chair, walk three meters, turn around, walk back to the chair, and sit down while performance was assessed in terms of time spent to complete the task. It has an excellent reliability, correlating in particular with gait speed and BI [35].

The 10-Meter Walking Test (10MWT) measures the time required to walk 10 meters, with the evaluation of walking speed (m/s) [35]. It has been used in various patient populations, including stroke [36]. Individuals were asked to walk at their preferred speed, also using assistive device.

The Six-Minute Walking Test (6MWT) was used to measure walking endurance, as usually described in many studies [37, 38]. Patients were asked to cover the maximum distance in six minutes at self-selecting speed. They were instructed that they could slow down and rest if necessary and then start again, if possible. Patients were allowed to use a cane or a walker and/or to perform the test under physiotherapist supervision or with a slight contact.

The Rivermead Mobility Index (RMI) was used to assess the mobility of patients. It is a scale measuring mobility of subjects with stroke in relationship to many aspects of their static and dynamic balance [39].

The Functional Ambulation Classification (FAC) is a functional 6-point walking scale to evaluate ambulation ability, assessing ambulation status regardless of patients' needing to use a personal assistive device [40]. FAC can be used with patients with stroke.

### 2.5. Statistical Analysis

Data were summarized in terms of their mean and standard deviation. *t*-test was used to compare age and time from event at baseline between the two groups. Parametric statistics, that is, repeated measure analysis of variance, was performed for continuous measures extracted by functional tests. Nonparametric statistics, as Friedman analysis for within subject comparisons along time (comparisons of data collected at T0, T1, T2 and T3) and Wilcoxon sign rank test for between subject comparisons (comparison of data of experimental group versus controlgroup). Threshold for statistical significance was set at 0.05. Independently by possible post hoc analyses a direct comparison of the changes occurred between the two groups in the period between T0 and T1 (value at T1-value at T0) using two-tailed Student *t*-test for continuous measures and Mann-Whitney *U* test for clinical ordinal scores. Correlation between these changes and T0 values was performed using Pearson and Spearman correlation coefficients for continuous and ordinal data, respectively.

## 3. Results

Of 115 screened patients admitted in our Unit in the 8 months of this study, fourteen patients matched inclusion/exclusion criteria and accepted to participate to the trial signing the informed consent and were hence enrolled into the study. These subjects were randomized into the experimental (*n* = 7) and control (*n* = 7) groups. Two patients of EG dropped out from the study (one at the first and the other one at the second session). Also one patient of control group dropped out for an emergency transfer to another hospital. Mean age of the sample of subjects who completed the protocol was 58.36 ± 14.35 years, with a time from stroke event of 19.09 ± 8.04 days, without any statistically significant differences between the groups (*P* = 0.137, *P* = 0.376, resp.). Demographical and clinical features of the enrolled patients are reported in Table 1.

**Figure 1:**
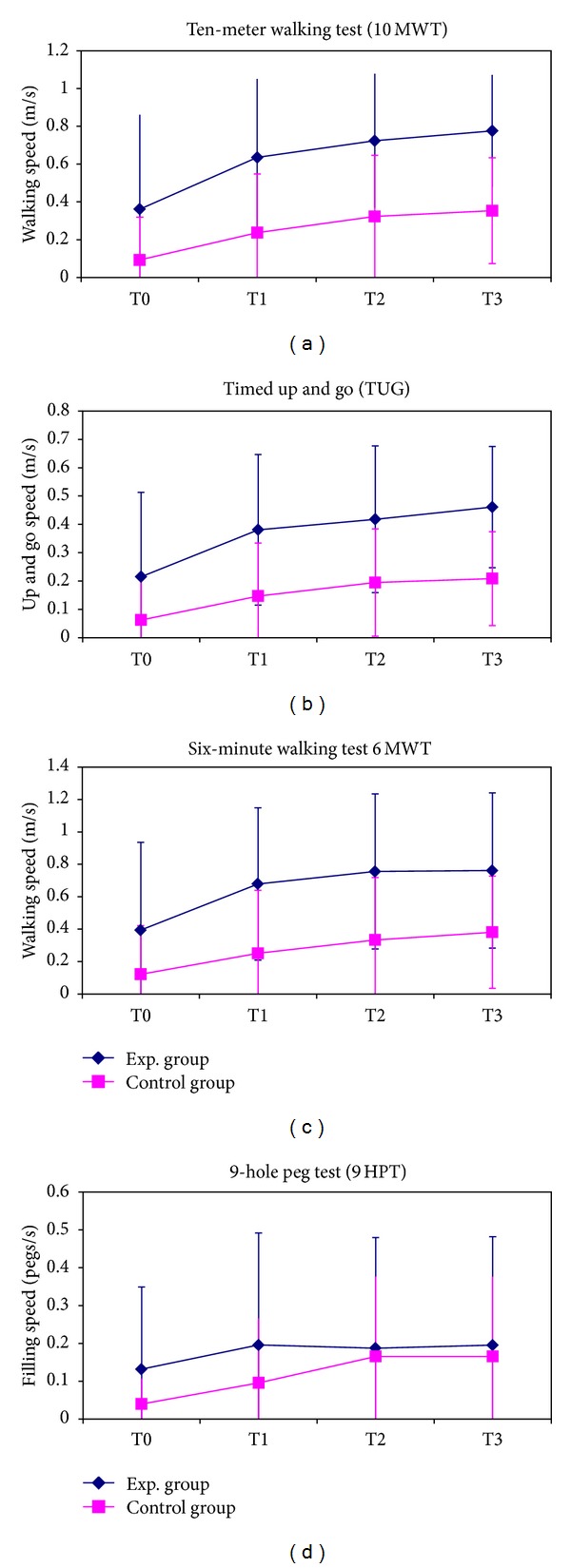
Mean and standard deviations for the functional tests.

The results of functional tests (10MWT, 6MWT, TUG, 9HPG) are reported in Figure 1.

Analysis of variance conducted on the eleven subjects who completed the study showed a statistically significant improvement along the rehabilitation, independently if experimental or conventional one, for the locomotor tests (10MWT, 6MWT, TUG) and for hand dexterity (9HPT), but not for hand force (neither pinch force, nor grasp force), as reported in Table 2. Some patients of both groups reported the insurgence of upper limb pain during rehabilitation and it limited the quantity of exercises performed for increasing hand forces.

**Figure 2:**
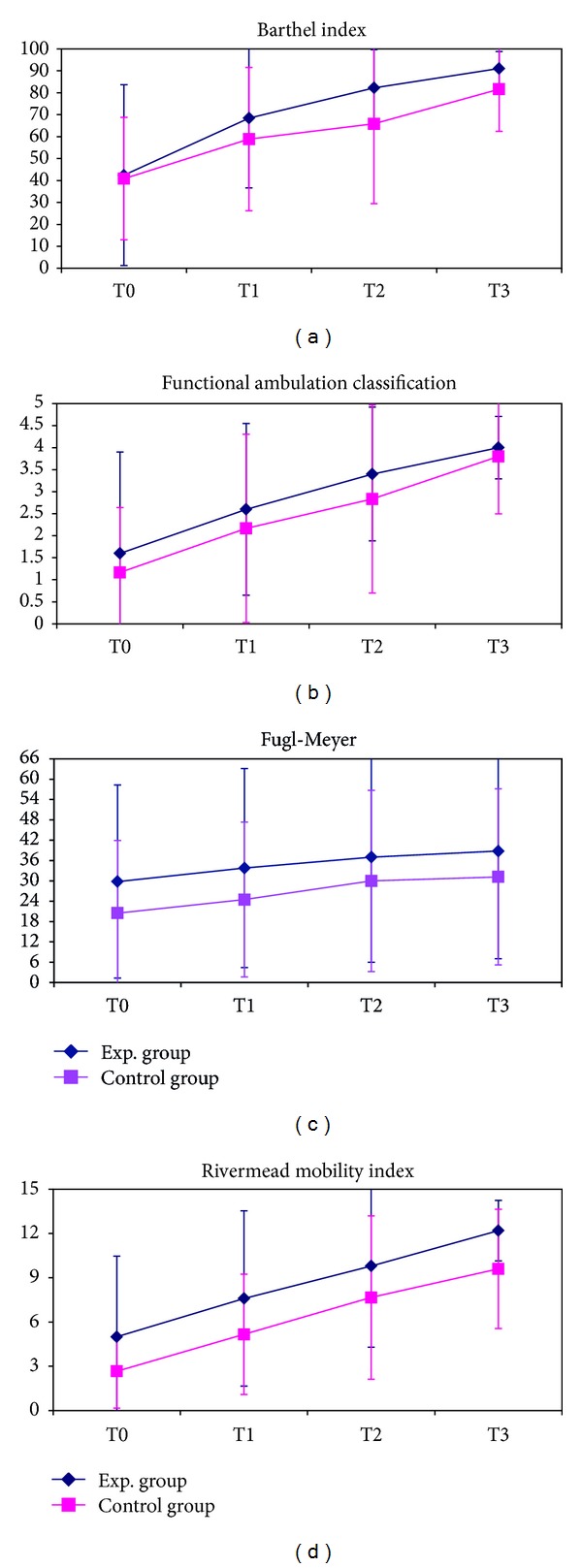
Mean and standard deviations for the clinical scores.

The results of clinical scales are shown in Figure 2. No significant differences were found between the two groups at baseline (T0) for BI-score (*P* = 0.931, Wilcoxon sign rank test), FAC-score (*P* = 0.931), CNS-score (*P* = 0.792), RMI-score (*P* = 0.537), and Fugl-Meyer-score (*P* = 0.444). Both groups showed significant improvements along rehabilitation as shown in Table 3, but without any statistically significant differences between groups assessed by Wilcoxon sign rank test.

In Table 4, the results of the comparisons of the changes occurring between T0 and T1 in the two groups are reported. No statistically significant differences were observed. About correlations between changes and T0-values, most of them were not statistically significantly and the trends were negative because T0 values were compared with T1 value-T0 value.

## 4. Discussion

The aim of this study was to determine whether a treatment with cathodal tDCS in adjunction to the traditional motor rehabilitation might improve the outcomes at the end of inpatient rehabilitation for patients affected by a first ever ischemic stroke in a subacute phase. To facilitate the spontaneous mechanisms of neuroplasticity, we developed this experimental protocol at an early phase of stroke rehabilitation (maximum within 30 days from the event). All the main features of motor impairments were assessed during the period of observation. Our results showed no difference in all these assessed domains of motor impairments between the experimental group and the control group.

Our trial provided stimulation at an early stage of the stroke rehabilitation, in order to favor the biological mechanisms of neuroplasticity involved in motor recovery. In fact, previous comparative preclinical studies have showed that tDCS could promote the same mechanisms involved in the neuroplastic reorganization [16]. The implementation of these systems has been suggested to be a promising factor in the recovery from stroke [41]. In a work of Vernieri and colleagues conducted on healthy subjects, it was observed that the tDCS could increase the blood flow in the brain below the stimulated area [42]. This increased flow was also present days away after the stimulation. The increased blood flow is considered an indicator of increased brain activity [43].

In the literature, the studies on the role of tDCS in stroke motor recovery are heterogeneous, depicting a fragmented framework. Some of them reported positive results also after a single session [44, 45], even if the most of these positive results concerned few weeks of stimulation [46, 47]. On the contrary, in the largest study on individuals with stroke in a rehabilitative setting, a combination of tDCS and robotic treatments in adjunction to motor rehabilitation was ineffective in producing functional improvements for different type of stroke lesions [48]. In that study, both anodal and cathodal stimulation were tested and no significant changes were reported for the most of assessed impairments. Only for cathodal tDCS, an improvement of the upper extremity functions in patients affected by lacunar stroke at the end of the stimulation was reported. Similarly, also our trial, conducted with a cathodal stimulation, did not show superior results in respect of conventional therapy alone. The absence of significant differences between the two groups of subjects involved in our study could be due to the reduced sample size. However, as shown in the figures, the trends of improvements resulted to be very similar between the two groups, and the consequent *P* values related to the interactions between treatment and time in the analysis of variance resulted far from the statistically significant threshold. It suggested that the absence of statistically significant differences was related to the similarity of the trends and probably not affected by the small sample size. Analogous results were found for anodal stimulation applied in the acute phase [49]. Our results seem to confirm this nonsuperior trend. On the contrary, these results contrast with others obtained in a previous trial by Fusco and colleagues in patients in a subacute phase of stroke, where an improvement of fine motor skills and manual force were founded [20].

A possible explanation for this lack of effect of tDCS in the stroke rehabilitation could be related to the severity of stroke and the degree of hemiparesis [50]. In their review, Schlaug and Renga noted that, in more affected patients, tDCS provided weak results in producing a noticeable effect, regardless of type of stimulation, due to the absence of intact tracts of pyramidal system [51]. In other experimental work, the same authors revealed that the cathodal stimulation could be the most suitable stimulation to produce an improvement of functional manual dexterity, even if they considered patients in chronic phase [52]. In our study, patients regained an improvement of the manual function not significantly different between the rehabilitation plus tDCS and only rehabilitation.

It has to be noted that our results differed from those of many other studies in the literature. For example, Kim and colleagues noted significant improvements both of simple and complex motor functions in the activities of daily living after a period of treatments with tDCS [21, 22]. This could be related to the fact that the enrolled patients were suffered subcortical lesions and often affected by hemorrhagic stroke, impacting positively on those results.

Analysing the impact of tDCS into rehabilitation of patients with stroke, too many studies were affected by important biases to give definitive indications. Many reviews and meta-analyses concluded suggesting caution into defining tDCS as an effective tool for improving neuromotor rehabilitation in stroke [17–19, 50]. In this sense, the disparate characteristics of enrolled patients with different types of stroke (cortical or cortical/subcortical or subcortical lesions; ischemic and hemorrhagic etiologies; acute, subacute, chronic phase; partial, total, or lacunar syndromes; different impairments at the enrollment of the trials) should be comprehended in a more homogeneous scenario in the future. Moreover, there is also the need of clear guidelines for the experimental use of tDCS to give exhaustive information about the potential role of this tool into stroke rehabilitation. In fact, use of different parameters of stimulation (including experimental setup, intensity, and current density) as well as the presence or absence of rehabilitative integration or the selection to adopt tDCS before or simultaneously with rehabilitation should be deeper analyzed.

Beyond the methodological considerations, our results about the nonuseful application of cathodal tDCS could be given by the results provided by Iosa and colleagues [24]. In this work, they showed that the use of the healthy upper limb may drive the improvement of the fine impairments of the paretic extremities, suggesting a bilateral transfer of motor skills probably related to the activities of mirror neurons involved in interhemispheric neurocomputational activities. The functional inhibition provided by cathodal tDCS in the not affected hemisphere could hamper this mechanism, limiting the recovery of manual force and dexterity.

At the moment, all these factors together do not allow a proper evaluation of the value of tDCS in a hypothetical rehabilitation protocol especially if compared to the extensive applications that tDCS has in other therapeutic fields, such as the psychiatric disorders [53].

Our results should be read at the light of the limitation of the study. In fact, the reduced sample size might have affected the power of statistical analyses bringing nonsignificant results. However, our sample size was in line with those of previous similar studies, and all of them highlighted the small number of enrolled subjects as a common limit of studies on cerebral direct current stimulation. To overcome this frequent limit, future studies should be designed as multicenter trials, involving wider and more homogeneous number of patients. For all these reasons, our study could be considered as a pilot. However, despite the reduced number of subjects and the high standard deviations affecting between-group comparisons, the trends of the improvement shown in Figures 1 and 2 resulted very similar between the two groups. Beyond the statistical meaning, the consistency of these trends among many different tests and clinical scales suggests the absence of any observable clinical differences between them. Other confounding factors could be present: the reduced period and the inadequate intensity of stimulation could be the reason for the low performance of tDCS in these patients. However, even if sessions of 10 minutes were demonstrated to be sufficient for eliciting prolonged after-effects for over an hour [15] and the current intensity is always enclosed between 1 and 2 mA [54, 55], the neurological deficits does not seem to benefit from the use of these parameters. Also the application of stimulation before the rehabilitative session could be tested in following studies. It has been hypothesized that the after-effects of tDCS are characterized by modification on NMDA receptors, but not on calcium channels [56]. This could reappraise the priming effects of tDCS, both for cathodal and anodal application [50]. Finally, few studies about tDCS in the neurologic rehabilitation are available to compare all the clinical scales we used. In fact, most of the works focus on segmental functions, while few studies focused on the global motor activities. Finally, the presence of pain which occurred to our patients during the inpatient rehabilitation could affect our results. At the same time, this symptom is quite common for this type of patient in subacute phase. In our study, pain arose in both groups, limiting it as confounding factor. Next studies should consider the presence of pain as possible bias.

To verify the present results, next clinical trials should consider the possibility of other types of stimulation (anodal or dual), in order to analyze different possible interactions between neuroplasticity and achievable motor outcomes, as well as a prolonged period of stimulation (i.e., for a total time of inpatient rehabilitation). In the future, as already reported for robotic-assisted rehabilitation [57], it will be crucial to identify which patients may be most likely to improve the rehabilitation benefiting by brain stimulation, especially in the long term [58], and which psychological features may affect the outcomes [59].

In conclusion, our study showed that an early applied cathodal electrical stimulation did not lead to a higher functional improvement in respect of traditional rehabilitation in patients affected by stroke in subacute phase during their period of inpatient rehabilitation.

## Figures and Tables

**Table 1:** Demographic and clinical characteristics of participants.

Pt. no.	Gender/Age	Group	Side affected	Handedness	Lesion	UL-FM	BI
1	F/63	CG	L	R	PACI	4	17
2	M/77	EG	R	L	TACI	22	26
3	M/77	CG	L	R	LACI	4	0
4	M/52	EG	R	L	TACI	4	8
5	F/33	EG	R	R	PACI	53	72
6	M/65	CG	R	R	PACI	52	65
7	F/51	EG	L	R	TACI	4	19
8	M/46	CG	L	R	TACI	17	53
9	F/69	EG	L	R	PACI	4	39
10	F/43	CG	R	R	PACI	66	100
11	F/66	CG	L	R	TACI	42	71

Pt.: patient, No.: number; ULFM: Upper Limb Fugl-Meyer; BI: Barthel index; M: male, F: female; EG: experimental group, CG: control group; R: right, L: left; Bamford Classification of stroke lesion: PACI: partial anterior circulation infarct, TACI: total anterior circulation infarct, LACI: lacunar infarct.

**Table 2:** Results of analysis of variance on functional tests.

Body structure and functioning	Tests	Within-group effect (T0, T1, T2, T3) (df = 3,27)	Between-group effects EG versus CG (df = 1,9)	Treatment effect (Interaction) (df = 3,27)
Lower limbs	10MWT	***P* < 0.001**	*P* = 0.078	*P* = 0.692
	6MWT	***P* = 0.001**	*P* = 0.149	*P* = 0.720
	TUG	***P* < 0.001**	*P* = 0.113	*P* = 0.601

Upper limbs	9HPT	***P* = 0.007**	*P* = 0.655	*P* = 0.372
	Pinch Force	*P* = 0.130	*P* = 0.612	*P* = 0.882
	Grasp Force	*P* = 0.990	*P* = 0.524	*P* = 0.672

df: degree of freedom of factor and related error (factor, error).

**Table 3:** Results of Friedman analysis along the rehabilitation period (T0, T1, T2, and T3) for the clinical scale scores.

Scale	Experimental group (df = 3)	Control group (df = 3)
BI	***P* = 0.012**	***P* = 0.001**
FAC	***P* = 0.015**	***P* = 0.008**
CNS	***P* = 0.004**	***P* = 0.005**
RMS	***P* = 0.010**	***P* = 0.002**
UL-FM	***P* = 0.045**	***P* = 0.003**

df: degree of freedom of the factor.

**Table 4:** Between group comparisons of changes occurred between T0 and T1.

Outcome measure	T1-T0 changes		Between group analysis *P* value (df = 1)	Correlation between changes and T0 values *R*-value	
	EG	CG		EG	CG
BI	26 ± 21	18 ± 10	0.584	−0.500	0.371
FAC	1 ± 1	1 ± 1	0.923	−0.530	0.557
CNS	1 ± 0.5	0.5 ± 1	0.219	−0.344	−0.223
RMS	3 ± 3	3 ± 2	0.853	0.205	0.696
UL-FM	4 ± 5	4 ± 7	0.925	0.026	0.585

10MWT (m/s)	0.27 ± 0.36	0.14 ± 0.23	0.485	−0.574	−0.071
6MWT (m/s)	0.28 ± 0.29	0.13 ± 0.31	0.419	−0.498	−0.197
TUG (m/s)	0.17 ± 0.19	0.08 ± 0.16	0.450	−0.389	−0.266
9HPT (pegs/s)	0.06 ± 0.09	0.06 ± 0.11	0.897	−0.476	0.886*
Pinch force (kg)	0.5 ± 1.4	0.8 ± 1.2	0.755	−0.130	0.464
Grasp force (kg)	−1.4 ± 1.9	1.0 ± 2.0	0.076	−0.922*	−0.250

Star indicates statistically significant correlation between changes and T0 value.
